# Regional cerebellum volume anomalies and associated cognitive function in children with fetal alcohol spectrum disorders

**DOI:** 10.1111/acer.70207

**Published:** 2025-11-28

**Authors:** Blake A. Gimbel, Donovan J. Roediger, Kent A. Tuominen, Mary E. Anthony, Alexandra L. Doughty, Abigail M. Ernst, Bryon A. Mueller, Erik de Water, the CIFASD, Jeffrey R. Wozniak

**Affiliations:** ^1^ Nationwide Children's Hospital Columbus Ohio USA; ^2^ The Ohio State University Columbus Ohio USA; ^3^ University of Minnesota Twin Cities Minneapolis Minnesota USA; ^4^ Great Lakes Neurobehavioral Center Eagan Minnesota USA

**Keywords:** cerebellum, cognition, fetal alcohol spectrum disorders, memory, prenatal alcohol exposure

## Abstract

**Background:**

Prenatal alcohol exposure (PAE) significantly impacts cerebellar development, which may affect cognitive function in fetal alcohol spectrum disorders (FASD). We evaluated cerebellar anomalies in children with FASD and an unexposed comparison group using an advanced MRI volumetric method that characterizes cerebellar structure at the level of individual lobules. We also explored associations between the cerebellum and cognitive performance.

**Methods:**

Forty‐seven children with FASD and 46 typically developing comparisons (ages 8–17 years). Participants completed a 3T MRI scan and cognitive testing including IQ, executive function, visual memory, and visual‐motor processing speed. T1‐weighted anatomical data were processed with the CerebNet pipeline, which segments the cerebellum into lobules. Univariate and multivariate linear models (with intracranial volume [ICV] as a covariate) examined group volume differences for total cerebellar volumes and individual cerebellar lobules. Exploratory analyses examined associations between cognitive functioning and cerebellum volumes.

**Results:**

Participants with FASD demonstrated lower volumes than comparison participants in total bilateral cerebellar white and cortical gray matter, and a smaller total vermis. Participants with FASD had lower regional volumes than comparisons within the bilateral lobules I–IV and V (anterior lobe) and vermis X lobule (flocculonodular lobe). Within the FASD group, a larger volume in the total cerebellar white matter was a significant predictor of better performance on measures of verbal working memory and processing speed. Within the comparison group, a larger volume of lobule V was a significant predictor of better verbal working memory, while a larger volume of lobule I–IV was a significant negative predictor of better verbal working memory.

**Conclusions:**

A novel MRI method to evaluate cerebellum morphology in children with FASD suggests that PAE is associated with cerebellum anomalies in a regionally specific manner. The data also suggest that structural anomalies of the cerebellum may have functional consequences for important cognitive skills in children with FASD.

## Graphical abstract

Prenatal alcohol exposure (PAE) significantly impacts cerebellum development, which may affect cognitive function in fetal alcohol spectrum disorders (FASD). Using an advanced MRI volumetric method that characterizes detailed cerebellum structure, this study identified regional cerebellum volume anomalies in children with FASD age 8–17 years. Examination of brain‐behavior relationships revealed cerebellum white matter volume as a significant predictor of cognitive function in several domains in FASD. Neurodevelopmental interventions targeting cerebellar development may be a promising avenue for supporting youth with FASD.

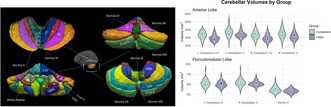

## INTRODUCTION

Prenatal alcohol exposure (PAE) can significantly disrupt brain development resulting in fetal alcohol spectrum disorders (FASD)—lifelong neurodevelopmental conditions associated with impairments in cognitive, behavioral and affective functioning (Popova et al., 2023). Globally estimated to affect 0.8 percent of the worldwide population (Popova et al., 2018), the prevalence of FASD in the United States is estimated at 2% to 5% (May et al., 2014). Individuals with FASD often have neurocognitive impairments in attention, executive functioning, memory, visual‐perceptual/motor skills and academic skills (Panczakiewicz et al., 2016). Neuroimaging identified structural brain anomalies in individuals with FASD including abnormalities in cortical thickness and cortical surface area, smaller whole‐brain and regional subcortical volumes, and atypical neurodevelopmental trajectories (Gimbel et al., 2024; Gimbel, Roediger, Anthony, et al., 2025; Moore & Xia, 2021). Among these neuroimaging findings, structural abnormalities have been identified in subcortical structures such as the hippocampus, caudate, and amygdala (Gimbel et al., 2024; Nardelli et al., 2011).

The cerebellum is well‐known to be involved in balance and motor control, but recent research has shown the cerebellum is also involved in many other cognitive and affective functions, including executive function, memory, language, and visuospatial processing (Schmahmann, 2019). A dense and anatomically complex structure, the cerebellum contains roughly 80 percent of the brain's neurons while occupying only 10 percent of the intracranial volume with a surface area equal to 78 percent of the cerebral cortex (Lyu et al., 2024). The cerebellum is anatomically divided into left and right hemispheres and the midline vermis (which is subdivided longitudinally into lobules). Each hemisphere is further subdivided into three distinct lobes composed of 10 individual lobules: the anterior lobe (lobules I–V), posterior lobe (lobules VI–IX), and the flocculonodular lobe (lobule X) (Stoodley & Schmahmann, 2018). Cerebellum development begins during the first 4 weeks after conception, making it one of the earliest structures within the central nervous system to form, which is in contrast to the cerebral cortex that experiences peak growth around 16 weeks post conception (Keefe & Nowakowski, 2020). The cerebellum initially arises from bilateral hindbrain tissues. The deep cerebellar nuclei form by the end of the first trimester, while granular layers (containing granule cells) and the midline vermis form during the second trimester. The volume of the cerebellum increases exponentially during gestation, with extensive axonal connections formed during the third trimester. Importantly, the cerebellum continues to mature well into the adulthood years (Olson et al., 2023).

With dense reciprocal neuronal loops connecting the cerebellum to the cerebral cortex, the cerebellum is implicated in a wide range of cognitive and emotional processes (Schmahmann, 2019). Studies examining the effects of cerebellar lesions and disruption with neuromodulation (e.g., with repetitive transcranial magnetic stimulation) implicate cerebellar dysfunction in impairments in executive function, attention, memory, and visuo‐spatial processing (Craig et al., 2021; Oliver et al., 2011; Shin et al., 2017; Zhang et al., 2023). Resting‐state and task‐based functional neuroimaging have identified functional specialization throughout the cerebellar cortex and its lobules as well as extensive cerebellar‐cortical co‐activation patterns (King et al., 2019; Sokolov et al., 2017). Atypical development of the cerebellum is implicated in a variety of congenital (e.g., Dandy Walker malformation, pontocerebellar hypoplasias) and neurodevelopmental conditions (e.g., autism spectrum disorder, attention‐deficit/hyperactivity disorder, and dyslexia) (Stoodley, 2014).

The complex and protracted development of the cerebellum makes it particularly vulnerable to developmental insults. Accumulating evidence from human and animal studies indicates the cerebellum is exceptionally vulnerable to the teratogenic insult of PAE (for review, see Leung et al., 2024). Animal models of PAE suggested the teratogenic insult on the cerebellum may result from diverse effects including oxidative stress, altered cell migration, and impaired growth signaling as several examples. Relevant to this study, several studies using animal models of PAE have documented volume and/or shape abnormalities in the cerebellum (e.g., Wang et al., 2020), although others have failed to find an effect on cerebellar morphology (e.g., Zhang et al., 2019). In humans, gross structural abnormalities of the cerebellum have been documented in both autopsy reports (Jarmasz et al., 2017) and prospective studies of individuals with FASD (Boateng et al., 2023; Boronat et al., 2017; Cardenas et al., 2014; Sullivan et al., 2020; Zhou et al., 2018). Older studies using manual tracing of the cerebellum suggested a particular impact of PAE on the anterior cerebellum (O'Hare et al., 2005; Sowell et al., 1996). Consistent with these findings, Cardenas et al. (2014), using an automated method to segment the cerebellum, further found smaller volumes in youth with PAE in the anterior (lobules I–V) and inferior posterior vermis (lobules VIII–X) but not the superior posterior vermis (lobules VI–VII) (Cardenas et al., 2014). Similarly, a vulnerability of anterior regions as well as the vermis (and less affected inferior and posterior cerebellar regions) was also documented by Fraize et al. (2023). In contrast, Sullivan et al. (2020) identified smaller posterior cerebellum volumes (lobules VIIB and VIIIA) in adolescents and young adults with fetal alcohol effects (an older term referring to individuals with PAE without meeting criteria for fetal alcohol syndrome [FAS]). They also found more widespread volume deficits including in the posterior lobe (lobules I–II, IV–VII, and VIII) in individuals with FAS. Several studies have also identified relationships between total cerebellar volumes and IQ (Boateng et al., 2023) as well as relationships between anterior vermal morphology and verbal memory performance (O'Hare et al., 2005) in individuals with PAE; however, another study found a positive correlation between cerebellar gray matter volume and verbal memory in controls but not those with PAE (Zhou et al., 2018). Together, these findings suggest that the growth and resulting volume of the cerebellum are disrupted in individuals with PAE and FASD, with some evidence for regionally specific deficits in the anterior region, although regional findings have been inconsistent. Such cerebellar dysfunction may also play an important role in neurocognitive functioning in individuals with FASD, and there is a clear need for further investigation.

Novel methods for characterizing cerebellum structure at the level of individual lobules provide opportunities for identifying regional volumetric anomalies in children with PAE that may have important implications for understanding cognitive dysfunction. Here, we extend previous work by using CerebNet (Faber et al., 2022), a novel automated tool that subsegments the cerebellum at the level of individual lobules, in order to further quantify cerebellar anomalies in children with PAE compared to unexposed children. This novel approach has important benefits over previous methods and has been shown to outperform other cerebellum subsegmentation tools, offering improved reliability and generalizability. By providing a detailed subsegmentation of individual cerebellar lobules as well as fine branching of cerebellar white matter that extend into the cerebellar cortex (an aspect of cerebellum structure that is ignored by other subsegmentation tools), this method provides valuable opportunities to characterize detailed structural anomalies in individuals with FASD. Additionally, we examine relationships between neurocognitive performance and cerebellum morphology. Because PAE has consistently been associated with brain growth anomalies (Donald et al., 2015), we hypothesized that both whole cerebellar (cortex and white matter) volumes and regional lobular volumes would be lower in youth with FASD compared to unexposed comparisons. We also hypothesized that smaller regional cerebellar volumes would be associated with lower performance on measures of global cognitive function, executive function, motor coordination, and memory.

## MATERIALS AND METHODS

### Participants

Participants included 93 children ages 8 to 16 years who were enrolled in the study as part of the Collaborative Initiative on Fetal Alcohol Spectrum Disorders (CIFASD) (see www.cifasd.org). The data used were from the University of Minnesota. From 2017 to 2019, participants were recruited by referral from the University of Minnesota Fetal Alcohol Spectrum Disorders Clinic and additional community postings, external clinics, and self‐referral. The child participants and their legal custodians completed the assent and consent processes on‐site, prior to data collection. Monetary compensation was provided for their involvement. The University of Minnesota Institutional Review Board approved all study procedures.

Exclusion criteria for both FASD and comparison groups included severe neurological or developmental disorders (e.g., autism spectrum disorder, schizophrenia, or epileptic seizures), extremely low birth weight (<1500 g), drug or alcohol misuse by the participant, and MRI contraindications. In the comparison group, prenatal substance exposure other than caffeine and tobacco was exclusionary; however, comorbid prenatal drug exposures in the PAE group were not exclusionary as prenatal polysubstance exposure is common (Popova et al., 2024).

### FASD diagnostic classification

Eligibility for enrollment was evaluated using a comprehensive phone screen and record review to determine history of PAE. Individuals within the FASD group were required to have a documented history of heavy PAE (≥13 drinks/week or ≥4 successive drinks during ≥1 week during pregnancy) unless diagnostic criteria were met for fetal alcohol syndrome (FAS) or partial fetal alcohol syndrome (PFAS) based on features relatively specific to PAE including facial dysmorphology and growth restriction. Physical assessments by trained investigators (Dr. Kenneth Lyons Jones or JRW) captured height, weight, occipitofrontal circumference (OFC), palpebral fissure length (PFL), and diagnostic ratings of the upper lip and philtrum. Diagnostic classification was completed in accordance with the Modified Institute of Medicine criteria for FASD (Hoyme et al., 2016). Growth deficiency was quantified as ≤10th percentile in height or weight for age and sex based on CDC Growth Charts (Grummer‐Strawn et al., 2010) and Nellhaus data were used to identify anomalies in OFC (Nellhaus, 1968). Neurobehavioral impairment was defined as global intellectual functioning ≥1.5 standard deviations (SD) below the mean (standard score ≤ 78) or ≥2 domains of impairment on individual cognitive measures. Neuropsychological tests and/or parent‐rated assessments were used to characterize neurobehavioral impairment.

### Neurobehavioral evaluation

Participants completed cognitive testing including measures of general intelligence (Full‐scale IQ [FSIQ]), visual‐motor processing speed, working memory, inhibitory control and cognitive flexibility, and visual memory. FSIQ and visual‐motor processing speed (processing speed index) were assessed using the Wechsler Intelligence Scale for Children, 5th Edition (WISC‐V). Working memory was assessed using the Wechsler Digit Span subtest and the NIH Toolbox List Sorting Working Memory Test (LST). Inhibitory control and cognitive flexibility were measured with the Delis‐Kaplan Executive Function System (D‐KEFS) Trail‐Making Test. Lastly, the NIH Toolbox Picture Sequence Memory Test (PSMT) was used to measure visual memory. Standard scores from these measures were converted to *z*‐score (*M* = 0, SD = 1) for consistency in comparison.

### MRI acquisition and processing

**FIGURE 1:**
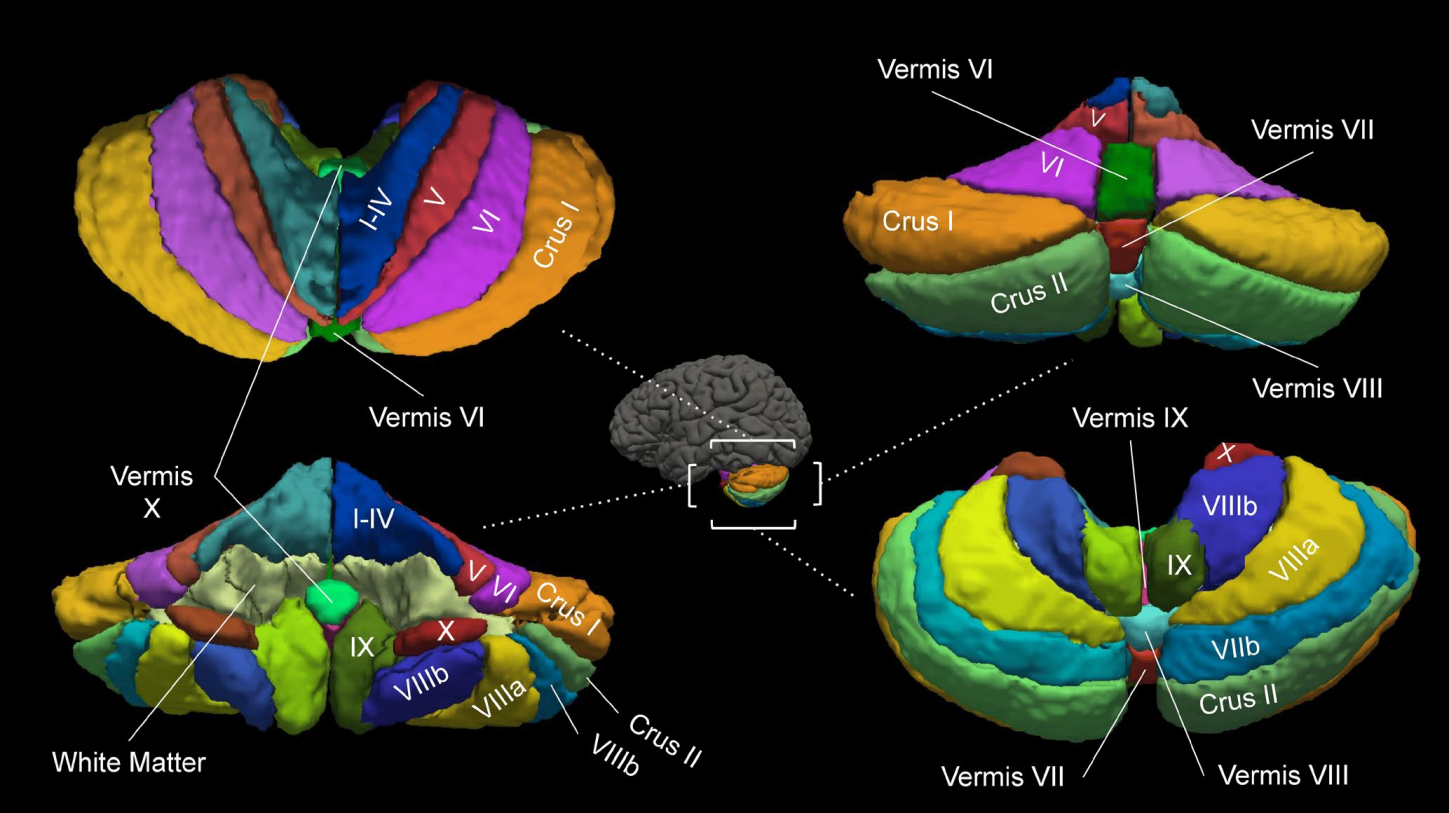
Three‐dimensional illustration of parcellation used to generate volumes for an individual study participant for separate lobules of the cerebellum. Clockwise from the top left: Dorsal view, posterior view, ventral view, anterior view.

A Siemens 3T Prisma scanner (Siemens, Erlangen, Germany) equipped with a standard 32‐channel head coil was used to acquire structural MRI data at the University of Minnesota's Center for Magnetic Resonance Research. Custom pulse sequences matching the Lifespan Human Connectome Project Development (HCP‐D) project were used to acquire T1‐weighted and T2‐weighted scans (Harms et al., 2018). This included automatic real‐time motion detection and k‐space line rejection and replacement software. T1‐weighted scans were acquired as follows: multiecho MP‐RAGE sequence with TR = 2500 ms, TE = 1.8/3.6/5.4/7.2 ms, TI = 1000 ms, voxel size = 0.8 mm isotropic, and flip angle = 8 degrees. The Euler value (Dale et al., 1999), averaged across hemispheres, was used to examine differences across diagnostic groups (FASD and comparison) in within‐scanner motion. Data were visually inspected by two trained operators (DJR, KAT) to ensure accuracy. The cerebellum was parcellated into lobules using unprocessed T1‐weighted images and CerebNet (Faber et al., 2022), a cerebellar segmentation pipeline included with the FastSurfer suite (Henschel et al., 2020) of deep‐learning‐based segmentation tools. Data from T2‐weighted scans were not included in the current analyses as they are not used by CerebNet. The CerebNet subsegmentation method provides detailed delineations in boundaries between cerebellar gray matter and cerebellar white matter including fine branches of white matter that extend into the cerebellar cortex. The automated tool provides a total of 30 regions of the cerebellum including 20 individual lobules (10 within each hemisphere), 5 vermis subsegments, 2 bilateral hemispheric gray matter volumes, 2 bilateral hemispheric white matter volumes, and 1 total vermal volume (Figure 1 shows one participant's parcellation as an example). Total volumes are also provided for each hemispheric cerebellar cortex and the vermis.

### Statistical analysis

Statistical analyses were performed using R version 4.1.1. Chi‐square and independent samples *t*‐tests were used to examine diagnostic group differences in demographic characteristics. Independent samples *t‐*tests were also used to examine group differences in within‐scanner motion (Euler values) and total intracranial volume (ICV). Analysis of covariance (ANCOVA; with ICV included as a covariate) examined group volume differences for total cerebellar hemisphere white and cortical gray matter, and total vermis. Multivariate analysis of covariance (MANCOVA; with ICV included as a covariate) examined group volume differences within each cerebellar lobe (anterior, posterior, flocculonodular). Significant main effects for group were further examined using ANCOVA (again with ICV included as a covariate) for each lobule. Given the number of comparisons of diagnostic group differences in cerebellar volumes (30 cerebellar regions), the false discovery rate was corrected using the Benjamini–Hochberg method (Benjamini & Hochberg, 1995) with *q* = 0.05. Corrected and uncorrected *p* values are reported. In an exploratory set of analyses, multiple linear regression (uncorrected for multiple comparisons) examined relationships between cognitive functioning and cerebellum volumes in regions in which group differences were found in order to estimate the unique contribution of each cerebellum volume to cognitive functioning. A total of six multiple regression models were run for each group (one for each of the six cognitive domains). Models were run separately for each group (FASD and comparison). Predictors (cerebellum volumes) were standardized separately within each group to improve comparability across variables (i.e., volumes were linearly transformed to *z*‐scores with a mean of 0 and standard deviation of 1). To reduce the number of comparisons and account for multicollinearity between cerebellar regions in each cerebellar hemisphere, cerebellar regions with bilateral group differences were summed across hemispheres. Lastly, we performed exploratory analyses to compare the Pearson's correlation coefficients for significant brain–behavior correlations (as identified in multiple regression analyses described above) across groups using Fischer *r*‐to‐*z* tests. Cerebellum volumes were again standardized separately within each group.

## RESULTS

### Subject characteristic

A total of 100 participants were enrolled in the study. Five participants in the comparison group were excluded for not completing an MRI. Two participants in the FASD group were excluded due to poor MRI data quality (based on visual inspection). This resulted in a total of 93 participants (47 FASD, 46 comparisons) included in the analyses. Demographic characteristics of the participants are summarized in Table 1. The average age of participants was not statistically different between the FASD group and the comparison group. Similarly, there were no significant differences in sex or ethnicity between the two groups. As expected, there was a statistically significant group difference in intelligence quotient (IQ) with the comparison group having a higher average IQ than the FASD group. There were statistically significant differences between the comparison and FASD groups in the proportions of Black, white, and multiracial participants. There were more Black and multiracial participants in the FASD group and more white participants in the comparison group. There were no other significant group differences in racial demographics. Handedness and growth deficiency were not statistically significant between the FASD group and the comparison group. Microcephaly and dysmorphic facial features had differences at the trend level—being more common in the FASD group. Finally, the majority of participants within the FASD group were diagnosed with ARND. The diagnosis of PFAS was the second most prevalent and the FAS diagnosis made up only 2% of the sample. There was no significant group difference in within‐scanner motion as estimated with Euler values, *t* (59) = 1.15, *p* = 0.253. Participants in the FASD group had significantly lower total intracranial volume (ICV; converted to cm^3^) than comparison participants (warranting including ICV as a covariate in subsequent analyses).

**TABLE 1:** Demographic characteristics of participants included in the analyses.

	FASD (*n* = 47)	Comparison (*n* = 46)	Statistical test
Age [*M* (SD)]	12.29 (2.36)	12.68 (2.60)	*t*(89) = 0.755, *p* = 0.452
Intelligence quotient [*M* (SD)]	93.11 (15.03)	115.44 (12.19)	*t*(88) = −7.847, *p* < 0.001
Intracranial volume cm^3^ [*M* (SD)]	1500.62 (137.42)	1593.40 (147.90)	*t*(88) = 3.11, *p* = 0.002
Sex [*n* (% female)]	25 (53%)	22 (47%)	𝝌^2^ = 0.096, *p* = 0.757
Ethnicity [*n* (%Hispanic)]	2 (4%)	3 (7%)	𝝌^2^ < 0.001, *p* = 0.980
Race			
[*n* (%American Indian/Alaska Native)]	4 (9%)	0 (0%)	𝝌^2^ = 2.280, *p* = 0.131
[*n* (%Asian)]	2 (4%)	1 (2%)	𝝌^2^ < 0.001, *p* = 1.000
[*n* (%Black or African American)]	7 (14%)	0 (0%)	𝝌^2^ = 5.420, *p* = 0.020
[*n* (%Native Hawaiian/Other Pacific Islander)]	1 (2%)	0 (0%)	𝝌^2^ < 0.001, *p* = 1.000
[*n* (%White)]	20 (43%)	44 (96%)	𝝌^2^ = 28.120, *p* < 0.001
[*n* (%Other)]	1 (2%)	0 (0%)	𝝌^2^ < 0.001, *p* = 1.000
[*n* (%Multiracial)]	13 (28%)	1 (2%)	𝝌^2^ = 9.900, *p* = 0.002
Handedness [*n* (%Right)]^a^	34 (79%)	39 (87%)	𝝌^2^ = 0.990, *p* = 0.610
Physical characteristics			
Growth deficiency^b^	6 (13%)	4 (9%)	𝝌^2^ = 0.090, *p* = 0.765
Microcephaly^c^	5 (11%)	0 (0%)	𝝌^2^ = 3.290, *p* = 0.070
Dysmorphic face^d^	11 (23%)	2 (4%)	𝝌^2^ = 7.560, *p* = 0.056
FASD diagnosis			
FAS [*n* (%FAS)]	1 (2%)	*NA*	
PFAS [*n* (%pFAS)]	11 (23%)	*NA*	
ARND [*n* (%ARND)]	35 (74%)	*NA*	

^a^ Handedness information was not available for 5 participants (4 FASD, 1 comparison). Two participants (1 FASD, 1 comparison) were ambidextrous.

^b^ Height or weight ≤10%ile.

^c^ Head circumference ≤10%ile.

^d^ At least two of the following: Palpebral fissure length ≤10%ile, thin vermillion border, smooth philtrum (4 or 5 on lipometer scale). The two comparison participants who had “dysmorphic faces” had scores of 4 on the philtrum and 4 on the vermillion border; neither had any other facial features or abnormal growth parameters.

### Group differences in total cerebellar volumes

Controlling for total ICV, there was a significant group effect, with participants in the FASD group demonstrating lower volumes than comparisons in the bilateral cerebellum white matter and cortex (Table 2). These group effects remained significant after FDR correction with the exception of the left cerebellum white matter, which was at the trend level. No significant group difference was observed in the vermis. Effect sizes were largest for the bilateral cerebellar cortex.

**TABLE 2:** Diagnostic group differences in total cerebellar volumes controlling for ICV.

Total volume	FASD		Comparison		*F*(1, 89)	*η* ^2^
	*M*	SD	*M*	SD		
L cerebellum white matter	11,717.02	1367.16	12,862.62	1476.79	*5.56* * ^,†^	0.027
R cerebellum white matter	11,549.06	1338.4	12,752.14	1444.79	7.68**	0.038
L cerebellum cortex	48,077.98	4744.08	52,695.59	5220.47	10.61**	0.062
R cerebellum cortex	47,908.95	4727.32	52,586.27	5343.73	10.63**	0.061
Vermis	5227.16	562.56	5654.52	637.56	3.60	0.024

*Note*: L = left hemisphere; R = right hemisphere; cerebellum volumes reported in mm^3^. Group differences that did not survive FDR correction but remained at the trend level (*p* = 0.05–0.07) are marked in italics and indicated with †.

* *p* < 0.05.

** *p* < 0.01.

### Group differences in individual cerebellar lobes and lobules

Controlling for total ICV, there was a significant group effect, with participants in the FASD group demonstrating lower volumes than comparisons in the anterior lobe, *F*(2, 89) = 6.62, *p* < 0.001, Pillai's trace = 0.236, partial *η* ^2^ = 0.24, and the flocculonodular lobe, *F*(2, 89) = 3.61, *p* = 0.016, Pillai's trace = 0.111, partial *η* ^2^ = 0.11. There was no effect of group for the posterior lobe, *F*(2, 89) = 1.49, *p* = 0.120, Pillai's trace = 0.271, partial *η* ^2^ = 0.27.

Given the significant group effect for the anterior and flocculonodular lobes, further univariate comparisons (again controlling for ICV) were conducted for individual lobules within each lobe. There was a significant group effect, with participants in the FASD group demonstrating lower volumes than comparisons, in the bilateral lobules I–IV and V and the vermis X (Table 3; Figure S1). After FDR correction, group differences in the bilateral lobules I–IV remained significant, and there were trend‐level group differences in the bilateral lobule V. A group difference in the vermis X did not survive FDR correction.

**TABLE 3:** Diagnostic group differences in cerebellar lobule volumes controlling for ICV.

Lobule volume	FASD		Comparison		*F*(1, 89)	*η* ^2^
	*M*	SD	*M*	SD		
Anterior lobe						
L cerebellum I–IV	2958.43	405.42	3289.49	456.13	7.40**	0.077
R cerebellum I–IV	2956.68	432.56	3323.48	467.66	8.55**	0.077
L cerebellum V	3340.31	414.46	3693.88	572.20	*5.97* * ^,^†	0.042
R cerebellum V	3162.64	471.12	3524.98	558.13	*5.72* * ^,^†	0.060
Flocculonodular lobe						
L cerebellum X	502.46	75.79	530.32	93.53	0.28	0.00
R cerebellum X	516.13	62.52	545.13	95.95	0.51	0.01
Vermis X	333.71	53.54	371.55	76.68	*4.42* *	0.05

*Note*: L = left hemisphere; R = right hemisphere; cerebellum lobule volumes reported in mm^3^. Group differences that did not survive FDR correction are marked in italic. Group differences that did not survive FDR correction but remained at the trend level (*p* = 0.05–0.07) are marked in italics and indicated with †.

* *p* < 0.05.

** *p* < 0.01.

### Relationship of cerebellum volumes to neurocognitive function

**FIGURE 2:**
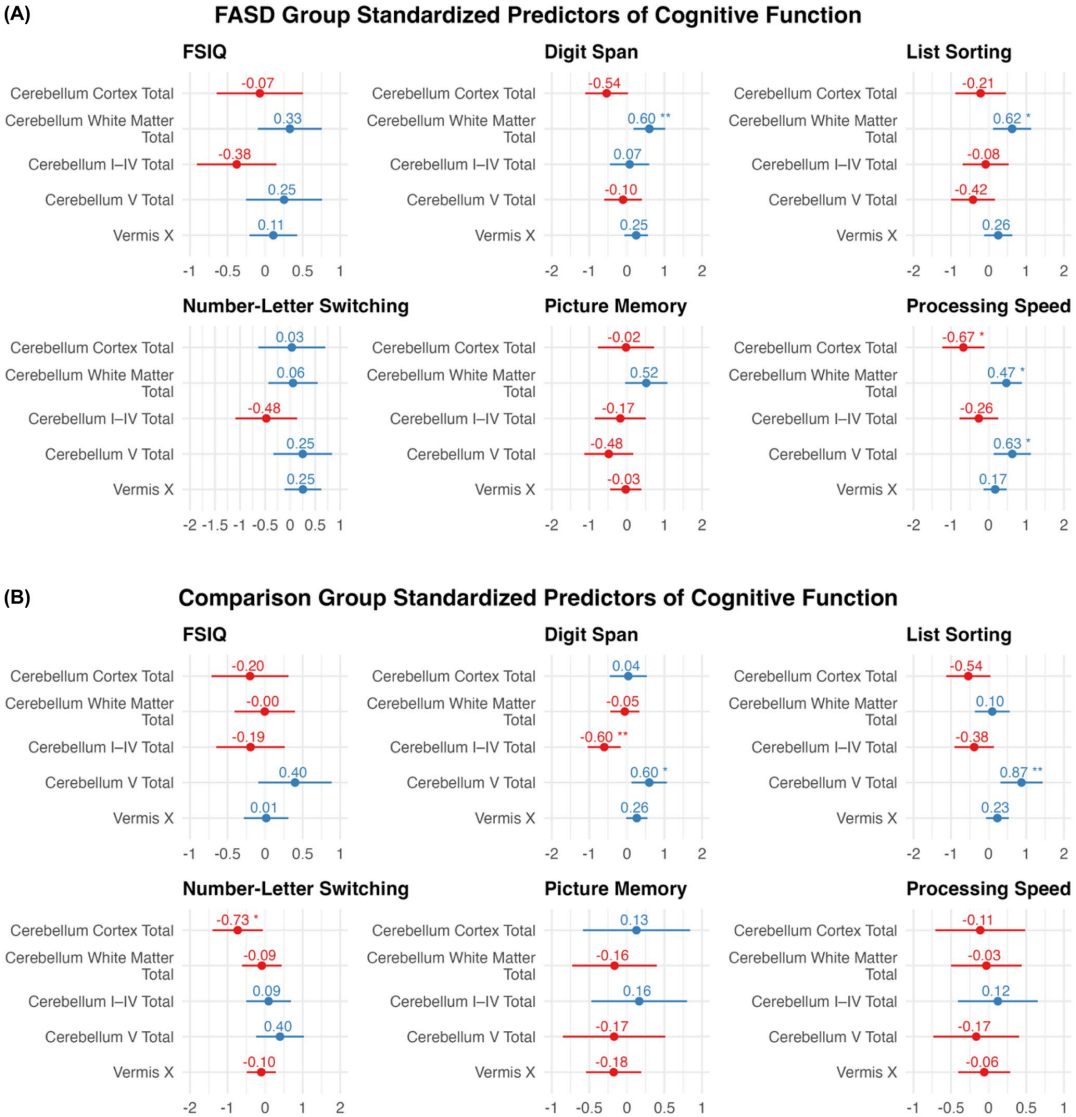
Unstandardized beta weights (*b*) from multiple linear regressions predicting cognitive function *z*‐scores in participants with FASD (A) and Comparisons (B). Predictors were standardized volumes of total cerebellar cortex, total cerebellar white matter, total lobules I–IV, total lobule V, and vermis X. Error bars represent 95% confidence intervals. * *p* < 0.05. **p < 0.01. Blue = positive association; Red = negative association.

Exploratory multiple linear regression analyses examining relationships between regional cerebellar volumes and neurocognitive function were performed separately for each group (Figure 2; Tables S1–S12).

*Within the FASD group*, cerebellum volumes significantly predicted verbal working memory (Wechsler Digit Span), *F*(5, 40) = 2.56, *p* = 0.042, explaining approximately 24% of the variance (*R* ^2^ = 0.24), and total cerebellar white matter volume was a significant predictor (*p* = 0.006) of better performance. Similarly, volumes predicted working memory (NIH Toolbox List Sorting), *F*(5, 40) = 2.84, *p* = 0.028, explaining approximately 26% of the variance (*R* ^2^ = 0.26), and total cerebellar white matter volume was again a significant predictor (*p* = 0.017) of better performance. Lastly, volumes predicted processing speed (Wechsler PSI), *F*(5, 41) = 3.56, *p* = 0.009, explaining approximately 30% of the variance (*R* ^2^ = 0.30). Significant predictors of better performance included volumes of the total cerebellar white matter (*p* = 0.027) and total lobule V (*p* = 0.014). The total cerebellar cortex was a significant predictor of worse performance (*p* = 0.020). Cerebellum volumes did not significantly predict IQ, inhibitory control and cognitive flexibility, or visual episodic memory in FASD participants.

*Within the comparison group*, cerebellum volumes significantly predicted verbal working memory (Wechsler Digit Span), *F*(5, 39) = 2.46, *p* = 0.049, explaining approximately 24% of the variance (*R* ^2^ = 0.24); total lobule V volume was a significant positive predictor (*p* = 0.014) while total lobule I‐IV volume was a significant negative predictor (*p* = 0.008). Similarly, volumes predicted verbal list‐sorting at a trend level (NIH Toolbox List Sorting), *F*(5, 40) = 2.24, *p* = 0.069, explaining approximately 22% of the variance in IQ (R^2^ = 0.22), and total lobule V volume was a significant predictor of better performance (*p* = 0.003). Cerebellum volumes did not significantly predict IQ, inhibitory control and cognitive flexibility, visual episodic memory, or processing speed in comparison participants.

Exploratory analyses comparing Pearson correlation coefficients (for significant brain‐behavior correlations identified in multiple regression analyses) indicated correlations were not significantly different across groups, *Z* scores ranged from −1.91 to 1.95, *p* values ranged from 0.052 to 0.949 (Table S13).

## DISCUSSION

In this study, we found volumetric anomalies in the cerebellum in children with FASD compared to typically developing, unexposed peers. Specifically, children with FASD have smaller cerebellar cortex volume and white matter volume than comparisons as well as smaller regional volumes within the anterior and flocculonodular lobes. This study extends previous research by using an advanced imaging analysis tool enabling detailed subsegmentation of individual cerebellar lobules as well as an examination of fine branching of cerebellar white matter that extends into the cerebellar cortex, which provides greater accuracy in quantifying cerebellar structure over older methods. We also identify FASD‐specific brain‐behavior relationships showing that regional cerebellum volumes are associated with cognitive function. Together, results provide further quantification of the lasting impact of PAE on the development of this important brain structure, which may meaningfully relate to functional outcomes for these individuals.

Participants with FASD demonstrated significantly smaller volumes than unexposed comparisons (even after controlling for expected group differences in intracranial volume) in total cerebellar cortex and white matter bilaterally, consistent with prior work showing gross structural anomalies in individuals with FASD (Boateng et al., 2023; Boronat et al., 2017; Cardenas et al., 2014; Sullivan et al., 2020; Zhou et al., 2018). Smaller white matter volumes more broadly have frequently been documented in previous imaging studies of FASD (Archibald et al., 2001; Donald et al., 2015; Treit et al., 2017). Notably, our findings show that PAE is associated with smaller cerebellar white matter volume even after controlling for intracranial volume. In other words, our data suggest that the cerebellum may be more impacted by PAE than other regions of white matter. Congruent with studies indicating that the anterior lobe is particularly vulnerable to the teratogenic effects of alcohol (Cardenas et al., 2014; Fraize et al., 2023), the FASD group demonstrated significantly lower volumes than comparisons in all lobules of the anterior lobe (lobules I‐IV and V bilaterally). The anterior lobe of the cerebellum has been hypothesized to play an important role in sensorimotor functions, with lesions producing dysmetria in the form of ataxic gait, limb dysmetria, and dysarthric speech (Schmahmann, 1991, 2019). Indeed, individuals with PAE/FASD have been shown to demonstrate impairments in motor coordination and gait abnormalities (Lucas et al., 2014) as well as speech/articulation deficits (Popova et al., 2016). We also observed significant group differences in the vermis X lobule of the flocculonodular lobe, aligning with the findings of Fraize et al. (2023) who documented smaller vermis volumes in individuals with FASD. The vermis has previously been shown to play an important role in cognitive function and motor skills. For example, a recent study using data from typically developing individuals from the Human Connectome Project (which also employed the CerebNet tool) found total vermis volume to be significantly associated with cognitive performance (Lutz et al., 2025). In addition, our FASD group showed no significant differences in the posterior lobe of the cerebellum, suggesting the posterior lobe may be relatively spared from structural abnormalities as a result of PAE. In contrast, Sullivan et al. (2020) identified smaller volumes in this region for children with FAS. This discrepancy may relate to our use of an advanced imaging analysis tool (CerebNet) as opposed to the older methods used by Sullivan et al. (2020) or differences in populations studied. The flocculonodular lobe is implicated in postural reflexes, balance, and oculomotor functions (Lara‐Aparicio et al., 2022; Ozgen et al., 2024). Notably, the midline vermis of the cerebellum (which extends across the anterior, posterior, and flocculonodular lobes) has been implicated specifically in limbic functions, with lesions shown to produce symptoms of cerebellar mutism and cerebellar cognitive affective syndrome (CCAS; Schmahmann, 2019). Our observation of smaller volumes of the vermis X lobule in individuals with FASD vs. comparisons is interesting in the context of previous work implicating specific vermal hypoplasia in individuals with FASD (Boronat et al., 2017) and animal studies showing a particular vulnerability of this region to alcohol teratogenesis (Sawant et al., 2013; Topper et al., 2015).

In exploratory analyses using multiple linear regression, divergent patterns of brain–behavior relationships emerged for each group. Notably, while Fisher's *r*‐to‐*z* comparisons of Pearson's correlation coefficients (i.e., between cerebellum volumes and cognitive measures by group) were not significant, multiple linear regression is preferable due to the ability to estimate the unique contribution of each cerebellar volume to cognitive performance while accounting for the variance in test scores attributable to other volumes. Specifically, in multiple regression models, a larger volume of the total cerebellum white matter emerged as a significant predictor of better performance on measures of verbal working memory and processing speed for participants in the FASD group. These findings are broadly consistent with previous non‐FASD research that showed deficits in executive function and visuospatial function with cerebellar lesions (see (Zhang et al., 2023) for review). However, our findings diverge from the previous non‐FASD research showing no significant relationship between lobular volume and impairments in IQ, inhibitory control, cognitive flexibility, and visual episodic memory (Craig et al., 2021; Oliver et al., 2011; Shin et al., 2017; Zhang et al., 2023). This discrepancy may stem from the difference in the cause of the cerebellum dysfunction. Many of these previous studies investigated the relationship between cerebellum integrity and cognition through the lens of acquired lesions (e.g., stroke). In contrast, our population experienced a very early neurodevelopmental insult and reduced subsequent cerebellar growth, which would be expected to be associated with a very different impact on cognitive function through both adaptive and maladaptive compensatory changes in neurodevelopment.

In contrast to FASD‐specific findings, within the comparison group, a larger volume of lobule V was a significant predictor of better verbal working memory, while a larger volume of lobule I‐IV was a significant predictor of worse performance. We also found that lobule V was a significant predictor of processing speed in FASD participants (measured in our study with visual‐motor processing speed tasks). This latter finding is consistent with prior work that has shown an association of the anterior cerebellum to motor tasks specifically. Together, lobules I‐V comprise the anterior lobe of the cerebellum. Lesion studies have associated anterior cerebellar damage with motor impairment (Stoodley et al., 2012). Consistent with this, in adults with multiple sclerosis lower anterior lobe volume was associated with worse fine motor performance (D'Ambrosio et al., 2017). Functional MRI has shown anterior regions to be primarily implicated in motor performance such as lateralized hand movements but also in divided attention and motor planning (King et al., 2019). Similarly, our finding that better verbal working memory correlated with larger lobule V volumes in comparison participants may suggest a role for anterior cerebellum regions in executive function performance. Our findings of worse verbal working memory performance with larger lobule I–IV volumes in comparison participants are more difficult to interpret and should be investigated further in future work.

The cerebellum has dense, intricate structural and functional connections with the cerebral cortex, brainstem, and spinal cord (as well as dense connections within and across the cerebellum itself), and it plays an important role in diverse neurocognitive functions (Schmahmann, 2019; Zhang et al., 2023). Therefore, the FASD‐specific findings here may reflect the degree to which the cerebellum and cerebral cortex are interconnected and interdependent during development [specifically, the interaction between the cerebellum and the prefrontal cortex (Allen et al., 2005)]. The prefrontal cortex is largely involved in executive functioning tasks such as inhibitory control, cognitive flexibility, and working memory. Moreover, a large number of studies of individuals with PAE have identified deficits in executive function and attention (Panczakiewicz et al., 2016). Importantly, a substantial proportion of variance in neurocognitive function was accounted for by cerebellar volumes in our findings (for example, cerebellar volumes accounted for 24%–30% of the variance in verbal working memory and processing speed performance in our FASD group). The fact that not all variance in executive function performance was predicted may relate to the different roles taken by the cerebellum and the cerebrum, or impacted communication between them. As such, other important factors (i.e., prefrontal and parietal networks) likely contribute substantially to functional deficits in individuals with FASD. Future research with larger sample sizes should examine structural and functional cerebral‐cerebellar networks that may be altered by PAE. In addition, given findings of atypical brain development across age from our own lab and others (Gimbel, Roediger, Ernst, Anthony, de Water, Mueller, et al., 2023; Gimbel, Roediger, Ernst, Anthony, de Water, Rockhold, et al., 2023; Moore & Xia, 2021), future research should also investigate atypical trajectories of cerebellar development in youth with FASD, perhaps by leveraging large‐scale normative data on cerebellar growth in typically developing children (Gaiser et al., 2024). Applying advanced tools to examine regional lobular volumes may provide a more detailed picture of age‐related developmental trajectories in this important structure.

Our finding of a prominent role for cerebellar white matter in predicting neurocognitive function in the FASD group aligns with a large body of evidence implicating white matter abnormalities resulting from PAE (Ghazi Sherbaf et al., 2019). Specifically, we observed a significant role for cerebellar white matter volume in accounting for performance on processing speed and working memory tasks in youth with FASD but not in comparison participants. Similarly, another study found a relationship between age‐related increases in white matter volume (e.g., in the inferior frontal white matter) and better cognitive function in individuals with FASD but not unexposed comparisons (Gautam et al., 2014). This finding could suggest a vulnerability to cerebellar‐cortical network‐level disruptions in FASD within the context of compromised white matter connectivity throughout the brain as a result of PAE. Previous works, including from our own lab, have observed significant correlations between cognitive performance and alterations in white matter development within the neocortex as a result of PAE (Ghazi Sherbaf et al., 2019; Gimbel et al., 2022; Gimbel, Roediger, Ernst, Anthony, de Water, Rockhold, et al., 2023). Indeed, previous work using diffusion tensor imaging (DTI) has demonstrated microstructural anomalies in the cerebellar peduncles (fiber pathways connecting the cerebellum to the brainstem) and associations with eye‐blink conditioning tasks (e.g., (Fan et al., 2015)) in individuals with PAE. Interestingly, a consistent finding across these three studies was a potentially lateralized teratogenic impact on the left middle cerebellar peduncle. Similarly, Lebel et al. (2010) identified a relationship between increased fractional anisotropy in the left middle cerebellar peduncle and math performance in youth with FASD (Lebel et al., 2010). In our study, we were limited to examining total volumes across cerebellar hemispheres due to our sample size and multicollinearity (a high correlation between left and right cerebellar volumes). Future work should further investigate regional and lateralized anomalies in cerebellar structure in relation to neurocognitive and neurobehavioral function in individuals with FASD. Together, the data presented here are compatible with and build upon previous findings identifying cerebellar white matter anomalies as an important target of PAE.

Given its vulnerability to teratogenic insult from PAE and its important role in diverse neurocognitive functions, the cerebellum may be an important target for interventions. For example, emerging research suggests neuromodulation of the cerebellum may be a promising approach to treating neurocognitive and neurobehavioral impairments in children with other neurodevelopmental conditions such as autism spectrum disorder (Elandaloussi et al., 2025). A large number of studies using animal models have investigated the role of choline and other agents in mitigating the effects of PAE on cerebellar development and function (see (Leung et al., 2024) for review and discussion). In our randomized controlled clinical trials using choline, an essential nutrient, as a neurodevelopmental intervention for children with FASD age 2–5 years, we have documented neurocognitive benefits that persist up to 7 years following intervention, which may reflect an impact on white matter microstructure (Gimbel et al., 2022). Neurodevelopmental interventions targeting cerebellar development may be a promising avenue for supporting youth with FASD.

### Limitations

This study has several important limitations to consider. The modest sample size may have limited our ability to detect meaningful group differences in cerebellar structure and differences in brain–behavior relationships in youth with FASD and unexposed comparisons. However, the data nevertheless revealed anomalies in cerebellar development in individuals with FASD, and our study benefited from an advanced tool to examine cerebellar structure in great detail. Importantly, given the exploratory nature of analyses examining relationships between cognitive functioning and cerebellum volumes, results were not corrected for multiple comparisons. Notably, although we focused on examining the relationship of cerebellar volumes to a range of cognitive functions, it is important to acknowledge that the cerebellum has also been strongly implicated in emotional and social functioning (Schmahmann & Sherman, 1998). Future research will benefit from examining the role of atypical cerebellum development resulting from PAE with regard to emotional and social domains, which are known to be impacted in youth with PAE and FASD (Mattson et al., 2019). An additional limitation in our sample was significant group differences (FASD vs. comparison) in race, which may limit the generalizability of our findings to other samples and populations. Importantly, previous research has identified associations between cerebellar structure and environmental factors such as stress and socioeconomic status (Kweon et al., 2022). As such, our findings of atypical cerebellum morphology in youth with FASD may represent the combined impact of a variety of environmental and psychosocial risk factors rather than the impact of PAE alone. Future research should replicate our findings in diverse populations with FASD and further examine the impact of these various factors on atypical brain development.

## CONCLUSIONS

We used an advanced neuroimaging tool to identify structural anomalies in the cerebellum in youth with FASD compared to unexposed peers. We also identified associations between cerebellar volumes and neurocognitive function with an important role of cerebellar white matter for youth with FASD.

## AUTHOR CONTRIBUTIONS

BAG: formal analysis, visualization, writing—original draft, writing—review and editing. DJR: investigation, data curation, formal analysis, methodology, visualization, writing—original draft, writing—review and editing. KAT: data curation. AME: writing—original draft, writing—review and editing. MEA: writing—original draft, writing—review and editing. ALD: writing—original draft, writing—review and editing. EdW: writing—original draft, writing—review and editing. BAM: investigation, data curation, formal analysis, methodology, visualization, writing—original draft, writing—review and editing. JRW: funding acquisition, project administration, resources, supervision, conceptualization, investigation, data curation, methodology, visualization, writing—original draft, writing—review and editing. All authors read and approved the final manuscript.

## FUNDING INFORMATION

All or part of this work was done in conjunction with the Collaborative Initiative on Fetal Alcohol Spectrum Disorders (CIFASD, https://doi.org/10.5967/ntw9‐h991), which is funded by grants from the National Institute on Alcohol Abuse and Alcoholism (NIAAA). Additional information about CIFASD, including information describing available data, can be found at www.cifasd.org. Support for this research was provided by the NIAAA (5U01AA026102, 5U24AA014815, 5U24AA014811), the National Institute of Biomedical Imaging and Bioengineering (NIBIB P41 EB027061), the NINDS (National Institute of Neurological Disorders and Stroke) (P41 EB015 894), the NINDS (National Institute of Neurological Disorders and Stroke) Institutional Center Core Grants to Support Neuroscience Research (P30 NS076408), and the National Institutes of Health, Office of the Director (1S10OD017974‐01).

## CONFLICT OF INTEREST STATEMENT

The authors have no conflicts of interest to disclose.

## ETHICS STATEMENT

All aspects of the study were approved by the University of Minnesota IRB and all participants' parents/guardians participated in a comprehensive informed consent procedure and signed consent forms.

## Supporting information


Figure S1

Appendix S1

## Data Availability

The datasets used and/or analyzed during the current study are available from the corresponding author upon reasonable request. Additional information can be found at cifasd.org.
