# Supplementary figures and images for: Regional cerebellum volume anomalies and associated cognitive function in children with fetal alcohol spectrum disorders
Source: Alcohol Clin Exp Res (Hoboken). 2025 Nov 28;50(1):e70207. doi: 10.1111/acer.70207 (PMC12668222; doi:10.1111/acer.70207)

# Cerebellar Volumes by Group

**A**

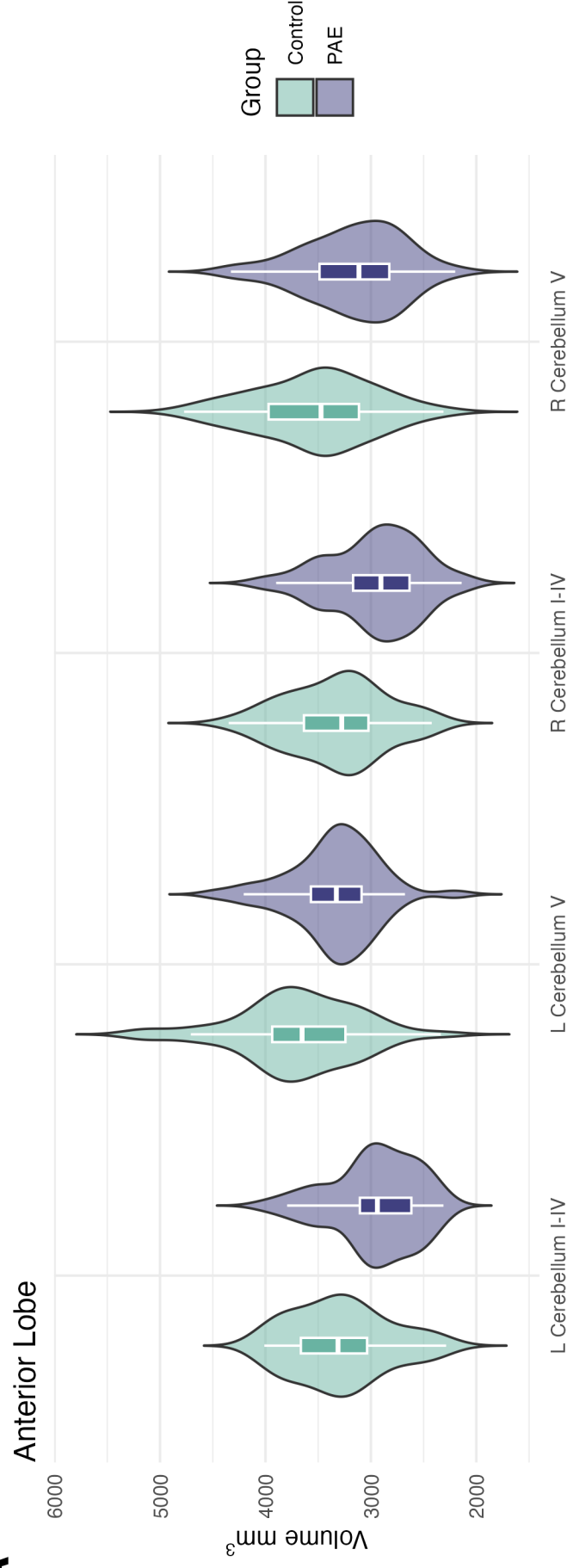

**B**

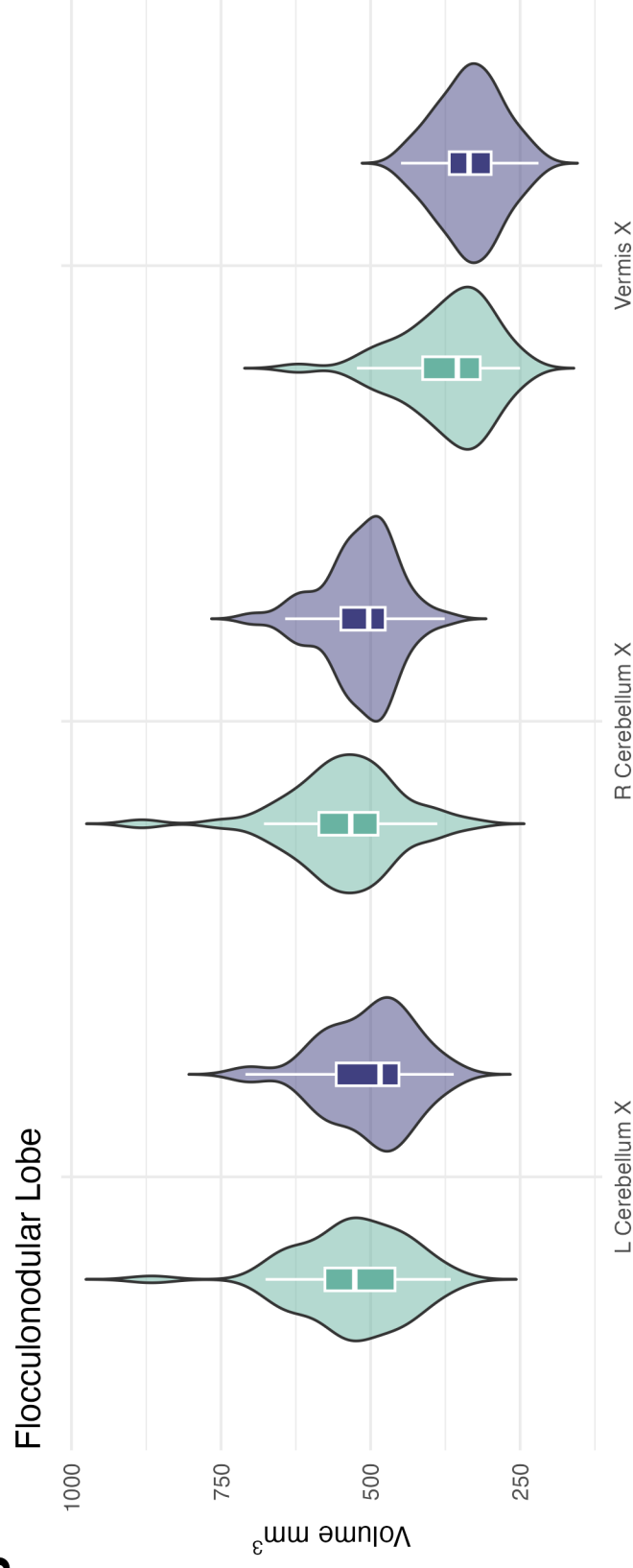

Supplement: Supplementary file 1 — Figure S1 [file ACER-50-0-s002.pdf]
