# Supplementary material for: Regional cerebellum volume anomalies and associated cognitive function in children with fetal alcohol spectrum disorders
Source: Alcohol Clin Exp Res (Hoboken). 2025 Nov 28;50(1):e70207. doi: 10.1111/acer.70207 (PMC12668222; doi:10.1111/acer.70207)
Supplement: Supplementary file 2 — Appendix S1 [file ACER-50-0-s001.docx]

Table S1: Regression results using Full_Scale_IQ_Composite_z as the criterion for the FASD group

| Predictor | *b* | *b*  95% CI  [LL, UL] | *beta* | *beta*  95% CI  [LL, UL] | *sr^2^* | *sr^2^*  95% CI  [LL, UL] | *r* | Fit |
| --- | --- | --- | --- | --- | --- | --- | --- | --- |
| (Intercept) | -0.46** | [-0.74, -0.18] |  |  |  |  |  |  |
| scale(Cerebellum_Cortex_Total) | -0.07 | [-0.64, 0.51] | -0.07 | [-0.64, 0.50] | .00 | [-.02, .02] | .11 |  |
| scale(Cerebellum_White_Matter_Total) | 0.33 | [-0.10, 0.75] | 0.33 | [-0.10, 0.75] | .05 | [-.06, .16] | .29* |  |
| scale(Cbm_I_IV_Total) | -0.38 | [-0.91, 0.15] | -0.38 | [-0.91, 0.15] | .04 | [-.06, .15] | -.08 |  |
| scale(Cbm_V_Total) | 0.25 | [-0.25, 0.76] | 0.25 | [-0.25, 0.76] | .02 | [-.05, .09] | .11 |  |
| scale(Cbm_Vermis_X) | 0.11 | [-0.21, 0.43] | 0.11 | [-0.21, 0.43] | .01 | [-.04, .06] | .22 |  |
|  |  |  |  |  |  |  |  | *R^2^*  = .171 |
|  |  |  |  |  |  |  |  | 95% CI[.00,.29] |
|  |  |  |  |  |  |  |  |  |

*Note.* A significant *b*-weight indicates the beta-weight and semi-partial correlation are also significant. *b* represents unstandardized regression weights. *beta* indicates the standardized regression weights. *sr^2^* represents the semi-partial correlation squared. *r* represents the zero-order correlation. *LL* and *UL* indicate the lower and upper limits of a confidence interval, respectively.
* indicates *p* < .05. ** indicates *p* < .01.

Table S2: Regression results using Working_Memory_Digit_Span_z as the criterion for the FASD group

| Predictor | *b* | *b*  95% CI  [LL, UL] | *beta* | *beta*  95% CI  [LL, UL] | *sr^2^* | *sr^2^*  95% CI  [LL, UL] | *r* | Fit |
| --- | --- | --- | --- | --- | --- | --- | --- | --- |
| (Intercept) | -0.50** | [-0.78, -0.21] |  |  |  |  |  |  |
| scale(Cerebellum_Cortex_Total) | -0.54 | [-1.11, 0.03] | -0.53 | [-1.08, 0.03] | .07 | [-.06, .20] | -.05 |  |
| scale(Cerebellum_White_Matter_Total) | 0.60** | [0.18, 1.02] | 0.59 | [0.18, 1.00] | .16 | [-.03, .34] | .27 |  |
| scale(Cbm_I_IV_Total) | 0.07 | [-0.45, 0.60] | 0.07 | [-0.44, 0.59] | .00 | [-.02, .02] | -.11 |  |
| scale(Cbm_V_Total) | -0.10 | [-0.60, 0.40] | -0.10 | [-0.59, 0.39] | .00 | [-.02, .03] | -.05 |  |
| scale(Cbm_Vermis_X) | 0.25 | [-0.06, 0.56] | 0.24 | [-0.06, 0.55] | .05 | [-.06, .16] | .25 |  |
|  |  |  |  |  |  |  |  | *R^2^*  = .242* |
|  |  |  |  |  |  |  |  | 95% CI[.00,.37] |
|  |  |  |  |  |  |  |  |  |

*Note.* A significant *b*-weight indicates the beta-weight and semi-partial correlation are also significant. *b* represents unstandardized regression weights. *beta* indicates the standardized regression weights. *sr^2^* represents the semi-partial correlation squared. *r* represents the zero-order correlation. *LL* and *UL* indicate the lower and upper limits of a confidence interval, respectively.
* indicates *p* < .05. ** indicates *p* < .01.

Table S3: Regression results using List_Sorting_z as the criterion for the FASD group

| Predictor | *b* | *b*  95% CI  [LL, UL] | *beta* | *beta*  95% CI  [LL, UL] | *sr^2^* | *sr^2^*  95% CI  [LL, UL] | *r* | Fit |
| --- | --- | --- | --- | --- | --- | --- | --- | --- |
| (Intercept) | -0.69** | [-1.02, -0.35] |  |  |  |  |  |  |
| scale(Cerebellum_Cortex_Total) | -0.21 | [-0.89, 0.46] | -0.18 | [-0.73, 0.37] | .01 | [-.04, .05] | -.03 |  |
| scale(Cerebellum_White_Matter_Total) | 0.62* | [0.12, 1.13] | 0.51 | [0.10, 0.93] | .11 | [-.05, .27] | .25 |  |
| scale(Cbm_I_IV_Total) | -0.08 | [-0.69, 0.53] | -0.07 | [-0.57, 0.44] | .00 | [-.02, .02] | -.22 |  |
| scale(Cbm_V_Total) | -0.42 | [-1.00, 0.17] | -0.34 | [-0.82, 0.14] | .04 | [-.06, .13] | -.19 |  |
| scale(Cbm_Vermis_X) | 0.26 | [-0.12, 0.63] | 0.21 | [-0.10, 0.51] | .03 | [-.06, .13] | .24 |  |
|  |  |  |  |  |  |  |  | *R^2^*  = .262* |
|  |  |  |  |  |  |  |  | 95% CI[.00,.39] |
|  |  |  |  |  |  |  |  |  |

*Note.* A significant *b*-weight indicates the beta-weight and semi-partial correlation are also significant. *b* represents unstandardized regression weights. *beta* indicates the standardized regression weights. *sr^2^* represents the semi-partial correlation squared. *r* represents the zero-order correlation. *LL* and *UL* indicate the lower and upper limits of a confidence interval, respectively.
* indicates *p* < .05. ** indicates *p* < .01.

Table S4: Regression results using Trails_Number_Letter_Switching_z as the criterion for the FASD group

| Predictor | *b* | *b*  95% CI  [LL, UL] | *beta* | *beta*  95% CI  [LL, UL] | *sr^2^* | *sr^2^*  95% CI  [LL, UL] | *r* | Fit |
| --- | --- | --- | --- | --- | --- | --- | --- | --- |
| (Intercept) | -0.43* | [-0.76, -0.09] |  |  |  |  |  |  |
| scale(Cerebellum_Cortex_Total) | 0.03 | [-0.63, 0.70] | 0.03 | [-0.56, 0.62] | .00 | [-.01, .01] | -.01 |  |
| scale(Cerebellum_White_Matter_Total) | 0.06 | [-0.44, 0.55] | 0.05 | [-0.39, 0.49] | .00 | [-.02, .02] | .09 |  |
| scale(Cbm_I_IV_Total) | -0.48 | [-1.09, 0.14] | -0.42 | [-0.97, 0.12] | .05 | [-.07, .17] | -.18 |  |
| scale(Cbm_V_Total) | 0.25 | [-0.33, 0.84] | 0.22 | [-0.30, 0.75] | .02 | [-.05, .08] | .01 |  |
| scale(Cbm_Vermis_X) | 0.25 | [-0.11, 0.62] | 0.23 | [-0.10, 0.55] | .04 | [-.07, .15] | .26 |  |
|  |  |  |  |  |  |  |  | *R^2^*  = .140 |
|  |  |  |  |  |  |  |  | 95% CI[.00,.25] |
|  |  |  |  |  |  |  |  |  |

*Note.* A significant *b*-weight indicates the beta-weight and semi-partial correlation are also significant. *b* represents unstandardized regression weights. *beta* indicates the standardized regression weights. *sr^2^* represents the semi-partial correlation squared. *r* represents the zero-order correlation. *LL* and *UL* indicate the lower and upper limits of a confidence interval, respectively.
* indicates *p* < .05. ** indicates *p* < .01.

Table S5: Regression results using Picture_Sequence_Memory_z as the criterion for the FASD group

| Predictor | *b* | *b*  95% CI  [LL, UL] | *beta* | *beta*  95% CI  [LL, UL] | *sr^2^* | *sr^2^*  95% CI  [LL, UL] | *r* | Fit |
| --- | --- | --- | --- | --- | --- | --- | --- | --- |
| (Intercept) | -0.27 | [-0.64, 0.10] |  |  |  |  |  |  |
| scale(Cerebellum_Cortex_Total) | -0.02 | [-0.77, 0.73] | -0.02 | [-0.59, 0.56] | .00 | [-.00, .00] | -.10 |  |
| scale(Cerebellum_White_Matter_Total) | 0.52 | [-0.05, 1.08] | 0.40 | [-0.04, 0.84] | .07 | [-.06, .20] | .13 |  |
| scale(Cbm_I_IV_Total) | -0.17 | [-0.85, 0.51] | -0.13 | [-0.66, 0.39] | .01 | [-.03, .04] | -.27 |  |
| scale(Cbm_V_Total) | -0.48 | [-1.13, 0.17] | -0.37 | [-0.87, 0.13] | .05 | [-.06, .15] | -.28 |  |
| scale(Cbm_Vermis_X) | -0.03 | [-0.45, 0.39] | -0.02 | [-0.34, 0.29] | .00 | [-.01, .01] | .00 |  |
|  |  |  |  |  |  |  |  | *R^2^*  = .192 |
|  |  |  |  |  |  |  |  | 95% CI[.00,.32] |
|  |  |  |  |  |  |  |  |  |

*Note.* A significant *b*-weight indicates the beta-weight and semi-partial correlation are also significant. *b* represents unstandardized regression weights. *beta* indicates the standardized regression weights. *sr^2^* represents the semi-partial correlation squared. *r* represents the zero-order correlation. *LL* and *UL* indicate the lower and upper limits of a confidence interval, respectively.
* indicates *p* < .05. ** indicates *p* < .01.

Table S6: Regression results using Processing.Speed.Composite_z as the criterion for the FASD group

| Predictor | *b* | *b*  95% CI  [LL, UL] | *beta* | *beta*  95% CI  [LL, UL] | *sr^2^* | *sr^2^*  95% CI  [LL, UL] | *r* | Fit |
| --- | --- | --- | --- | --- | --- | --- | --- | --- |
| (Intercept) | -0.31* | [-0.59, -0.04] |  |  |  |  |  |  |
| scale(Cerebellum_Cortex_Total) | -0.67* | [-1.23, -0.11] | -0.63 | [-1.16, -0.11] | .10 | [-.05, .25] | -.02 |  |
| scale(Cerebellum_White_Matter_Total) | 0.47* | [0.06, 0.89] | 0.44 | [0.05, 0.83] | .09 | [-.05, .23] | .26 |  |
| scale(Cbm_I_IV_Total) | -0.26 | [-0.78, 0.25] | -0.25 | [-0.73, 0.24] | .02 | [-.05, .08] | -.04 |  |
| scale(Cbm_V_Total) | 0.63* | [0.13, 1.12] | 0.59 | [0.13, 1.05] | .11 | [-.04, .27] | .22 |  |
| scale(Cbm_Vermis_X) | 0.17 | [-0.14, 0.48] | 0.16 | [-0.13, 0.45] | .02 | [-.05, .09] | .24 |  |
|  |  |  |  |  |  |  |  | *R^2^*  = .303** |
|  |  |  |  |  |  |  |  | 95% CI[.03,.43] |
|  |  |  |  |  |  |  |  |  |

*Note.* A significant *b*-weight indicates the beta-weight and semi-partial correlation are also significant. *b* represents unstandardized regression weights. *beta* indicates the standardized regression weights. *sr^2^* represents the semi-partial correlation squared. *r* represents the zero-order correlation. *LL* and *UL* indicate the lower and upper limits of a confidence interval, respectively.
* indicates *p* < .05. ** indicates *p* < .01.

Table S7: Regression results using Full_Scale_IQ_Composite_z as the criterion for the Comparison group

| Predictor | *b* | *b*  95% CI  [LL, UL] | *beta* | *beta*  95% CI  [LL, UL] | *sr^2^* | *sr^2^*  95% CI  [LL, UL] | *r* | Fit |
| --- | --- | --- | --- | --- | --- | --- | --- | --- |
| (Intercept) | 1.03** | [0.77, 1.28] |  |  |  |  |  |  |
| scale(Cerebellum_Cortex_Total) | -0.20 | [-0.71, 0.31] | -0.25 | [-0.88, 0.38] | .01 | [-.05, .08] | -.03 |  |
| scale(Cerebellum_White_Matter_Total) | -0.00 | [-0.41, 0.40] | -0.01 | [-0.50, 0.49] | .00 | [-.00, .00] | -.04 |  |
| scale(Cbm_I_IV_Total) | -0.19 | [-0.65, 0.26] | -0.24 | [-0.80, 0.32] | .02 | [-.06, .09] | -.03 |  |
| scale(Cbm_V_Total) | 0.40 | [-0.09, 0.89] | 0.49 | [-0.11, 1.10] | .07 | [-.07, .20] | .11 |  |
| scale(Cbm_Vermis_X) | 0.01 | [-0.28, 0.31] | 0.02 | [-0.31, 0.34] | .00 | [-.01, .01] | -.02 |  |
|  |  |  |  |  |  |  |  | *R^2^*  = .067 |
|  |  |  |  |  |  |  |  | 95% CI[.00,.14] |
|  |  |  |  |  |  |  |  |  |

*Note.* A significant *b*-weight indicates the beta-weight and semi-partial correlation are also significant. *b* represents unstandardized regression weights. *beta* indicates the standardized regression weights. *sr^2^* represents the semi-partial correlation squared. *r* represents the zero-order correlation. *LL* and *UL* indicate the lower and upper limits of a confidence interval, respectively.
* indicates *p* < .05. ** indicates *p* < .01.

Table S8: Regression results using Working_Memory_Digit_Span_z as the criterion for the Comparison group

| Predictor | *b* | *b*  95% CI  [LL, UL] | *beta* | *beta*  95% CI  [LL, UL] | *sr^2^* | *sr^2^*  95% CI  [LL, UL] | *r* | Fit |
| --- | --- | --- | --- | --- | --- | --- | --- | --- |
| (Intercept) | 0.31* | [0.07, 0.55] |  |  |  |  |  |  |
| scale(Cerebellum_Cortex_Total) | 0.04 | [-0.45, 0.53] | 0.04 | [-0.53, 0.61] | .00 | [-.01, .01] | .08 |  |
| scale(Cerebellum_White_Matter_Total) | -0.05 | [-0.44, 0.33] | -0.06 | [-0.51, 0.39] | .00 | [-.02, .02] | -.02 |  |
| scale(Cbm_I_IV_Total) | -0.60** | [-1.04, -0.16] | -0.70 | [-1.20, -0.19] | .15 | [-.03, .33] | -.10 |  |
| scale(Cbm_V_Total) | 0.60* | [0.13, 1.07] | 0.70 | [0.15, 1.24] | .13 | [-.04, .30] | .16 |  |
| scale(Cbm_Vermis_X) | 0.26 | [-0.02, 0.55] | 0.27 | [-0.02, 0.56] | .07 | [-.06, .20] | .19 |  |
|  |  |  |  |  |  |  |  | *R^2^*  = .240* |
|  |  |  |  |  |  |  |  | 95% CI[.00,.37] |
|  |  |  |  |  |  |  |  |  |

*Note.* A significant *b*-weight indicates the beta-weight and semi-partial correlation are also significant. *b* represents unstandardized regression weights. *beta* indicates the standardized regression weights. *sr^2^* represents the semi-partial correlation squared. *r* represents the zero-order correlation. *LL* and *UL* indicate the lower and upper limits of a confidence interval, respectively.
* indicates *p* < .05. ** indicates *p* < .01.

Table S9: Regression results using List_Sorting_z as the criterion for the Comparison group

| Predictor | *b* | *b*  95% CI  [LL, UL] | *beta* | *beta*  95% CI  [LL, UL] | *sr^2^* | *sr^2^*  95% CI  [LL, UL] | *r* | Fit |
| --- | --- | --- | --- | --- | --- | --- | --- | --- |
| (Intercept) | 0.09 | [-0.20, 0.37] |  |  |  |  |  |  |
| scale(Cerebellum_Cortex_Total) | -0.54 | [-1.13, 0.05] | -0.53 | [-1.10, 0.04] | .07 | [-.06, .20] | -.01 |  |
| scale(Cerebellum_White_Matter_Total) | 0.10 | [-0.37, 0.56] | 0.09 | [-0.36, 0.55] | .00 | [-.03, .03] | .01 |  |
| scale(Cbm_I_IV_Total) | -0.38 | [-0.91, 0.14] | -0.38 | [-0.89, 0.14] | .04 | [-.06, .15] | .03 |  |
| scale(Cbm_V_Total) | 0.87** | [0.31, 1.43] | 0.85 | [0.30, 1.40] | .19 | [-.01, .40] | .22 |  |
| scale(Cbm_Vermis_X) | 0.23 | [-0.07, 0.54] | 0.23 | [-0.07, 0.52] | .05 | [-.06, .16] | .15 |  |
|  |  |  |  |  |  |  |  | *R^2^*  = .219 |
|  |  |  |  |  |  |  |  | 95% CI[.00,.35] |
|  |  |  |  |  |  |  |  |  |

*Note.* A significant *b*-weight indicates the beta-weight and semi-partial correlation are also significant. *b* represents unstandardized regression weights. *beta* indicates the standardized regression weights. *sr^2^* represents the semi-partial correlation squared. *r* represents the zero-order correlation. *LL* and *UL* indicate the lower and upper limits of a confidence interval, respectively.
* indicates *p* < .05. ** indicates *p* < .01.

Table S10: Regression results using Trails_Number_Letter_Switching_z as the criterion for the Comparison group

| Predictor | *b* | *b*  95% CI  [LL, UL] | *beta* | *beta*  95% CI  [LL, UL] | *sr^2^* | *sr^2^*  95% CI  [LL, UL] | *r* | Fit |
| --- | --- | --- | --- | --- | --- | --- | --- | --- |
| (Intercept) | 0.13 | [-0.20, 0.46] |  |  |  |  |  |  |
| scale(Cerebellum_Cortex_Total) | -0.73* | [-1.40, -0.06] | -0.64 | [-1.22, -0.05] | .10 | [-.06, .26] | -.37* |  |
| scale(Cerebellum_White_Matter_Total) | -0.09 | [-0.61, 0.44] | -0.08 | [-0.54, 0.39] | .00 | [-.02, .03] | -.30* |  |
| scale(Cbm_I_IV_Total) | 0.09 | [-0.50, 0.69] | 0.08 | [-0.44, 0.60] | .00 | [-.02, .03] | -.17 |  |
| scale(Cbm_V_Total) | 0.40 | [-0.24, 1.04] | 0.35 | [-0.21, 0.91] | .03 | [-.06, .13] | -.14 |  |
| scale(Cbm_Vermis_X) | -0.10 | [-0.49, 0.29] | -0.08 | [-0.38, 0.22] | .01 | [-.03, .04] | -.13 |  |
|  |  |  |  |  |  |  |  | *R^2^*  = .202 |
|  |  |  |  |  |  |  |  | 95% CI[.00,.33] |
|  |  |  |  |  |  |  |  |  |

*Note.* A significant *b*-weight indicates the beta-weight and semi-partial correlation are also significant. *b* represents unstandardized regression weights. *beta* indicates the standardized regression weights. *sr^2^* represents the semi-partial correlation squared. *r* represents the zero-order correlation. *LL* and *UL* indicate the lower and upper limits of a confidence interval, respectively.
* indicates *p* < .05. ** indicates *p* < .01.

Table S11: Regression results using Picture_Sequence_Memory_z as the criterion for the Comparison group

| Predictor | *b* | *b*  95% CI  [LL, UL] | *beta* | *beta*  95% CI  [LL, UL] | *sr^2^* | *sr^2^*  95% CI  [LL, UL] | *r* | Fit |
| --- | --- | --- | --- | --- | --- | --- | --- | --- |
| (Intercept) | 0.77** | [0.42, 1.12] |  |  |  |  |  |  |
| scale(Cerebellum_Cortex_Total) | 0.13 | [-0.59, 0.84] | 0.11 | [-0.52, 0.75] | .00 | [-.03, .04] | -.04 |  |
| scale(Cerebellum_White_Matter_Total) | -0.16 | [-0.73, 0.40] | -0.15 | [-0.65, 0.36] | .01 | [-.04, .06] | -.09 |  |
| scale(Cbm_I_IV_Total) | 0.16 | [-0.47, 0.80] | 0.15 | [-0.42, 0.72] | .01 | [-.04, .05] | -.02 |  |
| scale(Cbm_V_Total) | -0.17 | [-0.85, 0.51] | -0.15 | [-0.76, 0.46] | .01 | [-.04, .05] | -.05 |  |
| scale(Cbm_Vermis_X) | -0.18 | [-0.54, 0.19] | -0.16 | [-0.48, 0.17] | .02 | [-.06, .11] | -.14 |  |
|  |  |  |  |  |  |  |  | *R^2^*  = .035 |
|  |  |  |  |  |  |  |  | 95% CI[.00,.06] |
|  |  |  |  |  |  |  |  |  |

*Note.* A significant *b*-weight indicates the beta-weight and semi-partial correlation are also significant. *b* represents unstandardized regression weights. *beta* indicates the standardized regression weights. *sr^2^* represents the semi-partial correlation squared. *r* represents the zero-order correlation. *LL* and *UL* indicate the lower and upper limits of a confidence interval, respectively.
* indicates *p* < .05. ** indicates *p* < .01.

Table S12: Regression results using Processing.Speed.Composite_z as the criterion for the Comparison group

| Predictor | *b* | *b*  95% CI  [LL, UL] | *beta* | *beta*  95% CI  [LL, UL] | *sr^2^* | *sr^2^*  95% CI  [LL, UL] | *r* | Fit |
| --- | --- | --- | --- | --- | --- | --- | --- | --- |
| (Intercept) | 0.67** | [0.37, 0.96] |  |  |  |  |  |  |
| scale(Cerebellum_Cortex_Total) | -0.11 | [-0.71, 0.49] | -0.12 | [-0.76, 0.52] | .00 | [-.03, .04] | -.20 |  |
| scale(Cerebellum_White_Matter_Total) | -0.03 | [-0.50, 0.44] | -0.03 | [-0.54, 0.47] | .00 | [-.01, .01] | -.16 |  |
| scale(Cbm_I_IV_Total) | 0.12 | [-0.41, 0.65] | 0.13 | [-0.44, 0.70] | .01 | [-.04, .05] | -.13 |  |
| scale(Cbm_V_Total) | -0.17 | [-0.74, 0.40] | -0.18 | [-0.79, 0.44] | .01 | [-.04, .06] | -.19 |  |
| scale(Cbm_Vermis_X) | -0.06 | [-0.40, 0.29] | -0.06 | [-0.38, 0.27] | .00 | [-.03, .03] | -.07 |  |
|  |  |  |  |  |  |  |  | *R^2^*  = .050 |
|  |  |  |  |  |  |  |  | 95% CI[.00,.10] |
|  |  |  |  |  |  |  |  |  |

*Note.* A significant *b*-weight indicates the beta-weight and semi-partial correlation are also significant. *b* represents unstandardized regression weights. *beta* indicates the standardized regression weights. *sr^2^* represents the semi-partial correlation squared. *r* represents the zero-order correlation. *LL* and *UL* indicate the lower and upper limits of a confidence interval, respectively.
* indicates *p* < .05. ** indicates *p* < .01.

Table S13: Fisher’s r-to-z comparison of Pearson correlation coefficients between cerebellum volumes and cognitive measures by group

| Cerebellum Region | Cognitive Measure | *r* (FASD) | n (FASD) | *r* (Comparison) | n (Comparison) | Z | p (Diff) |
| --- | --- | --- | --- | --- | --- | --- | --- |
| Cerebellum Cortex Total | Processing Speed | -0.02 | 47 | -0.20 | 45 | 0.85 | 0.394 |
| Cerebellum Cortex Total | Number-Letter Switching | -0.01 | 46 | -0.37 | 45 | 1.74 | 0.081 |
| Cerebellum White Matter Total | Digit Span | 0.27 | 46 | -0.02 | 45 | 1.37 | 0.170 |
| Cerebellum White Matter Total | List Sorting | 0.25 | 46 | 0.01 | 46 | 1.14 | 0.255 |
| Cerebellum White Matter Total | Processing Speed | 0.26 | 47 | -0.16 | 45 | 1.94 | 0.052 |
| Cerebellum I-IV Total | Digit Span | -0.11 | 46 | -0.10 | 45 | -0.06 | 0.949 |
| Cerebellum V Total | Processing Speed | 0.22 | 47 | -0.19 | 45 | 1.95 | 0.052 |
| Cerebellum V Total | Digit Span | -0.05 | 46 | 0.16 | 45 | -0.97 | 0.330 |
| Cerebellum V Total | List Sorting | -0.19 | 46 | 0.22 | 46 | -1.91 | 0.056 |

Figure S1: Violin plots displaying cerebellar regional volumes by group in regions in which significant group differences were observed
